# 7,7-Dimethyl-3,3,4a-tris­(3-methyl­but-2-en­yl)-4a,5,6,7-tetra­hydro-2*H*-chromene-2,4(3*H*)-dione

**DOI:** 10.1107/S1600536809025021

**Published:** 2009-07-04

**Authors:** Katrin Möws, Markus Schürmann, Hans Preut, Bernd Plietker

**Affiliations:** a Fakultät Chemie, Technische Universität Dortmund, Otto-Hahn-Strasse 6, 44221 Dortmund, Germany; b Institut für Organische Chemie, Fakultät Chemie, Universität Stuttgart, Pfaffenwaldring 55, 70569 Stuttgart, Germany

## Abstract

The title compound, C_26_H_38_O_3_, was prepared by an intra­molecular Claisen-like cyclization of ethyl 2-acet­oxy-4,4-dimethyl-1-(3-methyl­but-2-en­yl)cyclo­hex-2-enecarboxyl­ate followed by dialkyl­ation. One of the methyl groups is disordered over two sets of sites in a 0.67:0.33 ratio.

## Related literature

For further information, see: Ciochina & Grossman (2006 ▶).
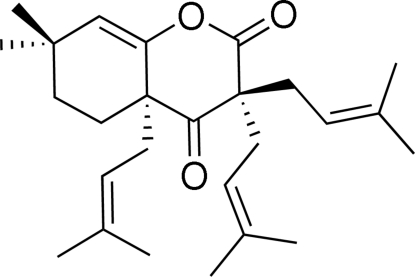


## Experimental

### 

#### Crystal data

C_26_H_38_O_3_
*M* *_r_* = 398.56
Triclinic, 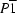

*a* = 9.534 (2) Å
*b* = 11.753 (3) Å
*c* = 11.994 (2) Å
α = 77.862 (13)°
β = 78.325 (12)°
γ = 66.427 (9)°
*V* = 1193.7 (5) Å^3^
*Z* = 2
Mo *K*α radiation
μ = 0.07 mm^−1^
*T* = 173 K
0.50 × 0.27 × 0.04 mm

#### Data collection

Nonius KappaCCD diffractometer
Absorption correction: none
14072 measured reflections
4134 independent reflections
1323 reflections with *I* > 2σ(*I*)
*R*_int_ = 0.078

#### Refinement

*R*[*F* ^2^ > 2σ(*F* ^2^)] = 0.036
*wR*(*F* ^2^) = 0.112
*S* = 1.02
4134 reflections
280 parameters
H-atom parameters constrained
Δρ_max_ = 0.11 e Å^−3^
Δρ_min_ = −0.10 e Å^−3^

### 

Data collection: *COLLECT* (Nonius, 1998 ▶); cell refinement: *DENZO* and *SCALEPACK* (Otwinowski & Minor, 1997 ▶); data reduction: *DENZO* and *SCALEPACK*; program(s) used to solve structure: *SHELXS97* (Sheldrick, 2008 ▶); program(s) used to refine structure: *SHELXL97* (Sheldrick, 2008 ▶); molecular graphics: *SHELXTL-Plus* (Sheldrick, 2008 ▶); software used to prepare material for publication: *SHELXL97* and *PLATON* (Spek, 2009 ▶).

## Supplementary Material

Crystal structure: contains datablocks I, global. DOI: 10.1107/S1600536809025021/hb5012sup1.cif

Structure factors: contains datablocks I. DOI: 10.1107/S1600536809025021/hb5012Isup2.hkl

Additional supplementary materials:  crystallographic information; 3D view; checkCIF report

## Figures and Tables

**Fig. 1.**
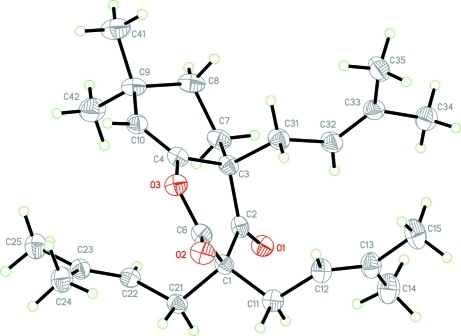
: The molecular structure of (I) with displacement ellipsoids shown at the 30% probability level. Of the two disordered positions (C35 and C35') only one is shown.

## supplementary crystallographic information

### Experimental

Diethyl ether was dryed over sodium. All other solvents and reagents were commercially available and used as received. Flash-chromatography was performed on silicagel 60 (230–400 mesh) using head pressure by means of compressed air. Infrared spectra (IR) were recorded as a thin film between KBr-plates. The instrument used was a Bruker IFS 66 FT—IR spectrophotometer. GC—MS spectra were recorded on a Finnigan Polaris GCQ spectrometer. Proton (^1^H NMR, 500 MHz) and carbon (^13^C NMR, 125 MHz) nuclear magnetic resonance spectra were recorded in chloroform(d-1) and referenced to the solvent signal. The instrument used was a Bruker DRX 500. The multiplicities of the signals are given as s (singlet), d (doublet), t (triplet), and m (multiplet).

HMDS (780 µ*L*, 3.425 mmol) was dissolved in diethylether (3 ml) at 273 K. Butyllithium (2.1 ml, 3.36 mmol, 1.6 *M* in hexane) was added at that temperature and the mixture was stirred for 15 minutes. After cooling to 195 K a suspension of CuI (360 mg, 1.89 mmol) and ethyl 2-acetoxy-4,4-dimethyl-1-(3-methylbut-2-enyl)cyclohex-2-enecarboxylate (580 mg, 1.88 mmol) in diethylether (3 ml) was added (Ciochina & Grossman, 2006). The mixture was stirred for 2 h. Isoprenylbromide (440 µl, 3.78 mmol) was added dropwise and the mixture was stirred for 5 days at room temperature. An aqueous Seignette salt-solution was added. Phases were separated and the aqueous layer was extracted with diethylether (3 *x* 10 ml). The combined organic layers were dried over Na_2_SO_4_ and concentrated in vacuum. The crude product was purified *via* column chromatography (20:1 i-hexane/ethyl acetate). The title compound was obtained in 30% yield (223 mg, 0.559 mmol) and crystallized by slow evaporation of a mixture of diethyether and i-hexane.

*R*_f_: 0.57 (isohexan/ethyl acetate 10:1), mp: 325 K, ^1^H NMR (500 MHz, CDCl_3_): δ(p.p.m.) = 5.40 (s, 1H, CH), 5.01–4.96 (m, 2H, CH), 4.84 (m, 1H, CH–), 2.64–2.54 (m, 3H, CH_2_), 2.33–2.26 (m, 2H, CH_2_), 2.07–2.00 (m, 2H, CH_2_), 1.82–1.87 (m, 1H, CH_2_), 1.68 (s, 3H, CH_3_), 1.63 (s, 3H, CH_3_), 1.57/1.58 (2*s, 9H, CH_3_), 1.46–1.41 (m, 1H, CH_2_), 1.26–1.21 (m, 1H, CH_2_), 1.03 (s, 3H, CH_3_), 1.00 (s, 3H, CH_3_). ^13^C NMR (125 MHz, CDCl_3_): δ (p.p.m.) = 206.6, 169.7, 146.0, 137.1, 135.9, 135.6, 122.3, 117.5, 116.6, 61.5, 50.3, 37.4, 33.9, 32.4, 32.2, 29.7, 29.2, 26.2, 26.1, 26.0, 24.8, 18.2, 18.0. IR (film): ν (cm^-1^) = 2963 (*m*), 2917 (*m*), 1770 (*s*), 1717 (*s*), 1674 (*m*), 1449 (*m*), 1221 (*m*), 1113 (*m*), 1062 (*m*). MS (EI, 70 eV): m / *z* (%) = 398 (2) [*M*^+^], 370 (1) [C_25_H_38_O2^+^], 329 (28) [C_21_H_29_O_3_^+^], 315 (16) [C_21_H_31_O_2_^+^], 301 (1) [C_25_H_38_O_2_^+^], 262 (100) [C_16_H_22_O_3_^+^], 247 (69), 206 (77) [C_13_H_18_O_2_^+^], 191 (57) [C_12_H_15_O_2_^+^], 177 (7) [C_12_H_17_O^+^], 137 (10) [C_9_H_13_O^+^], 69 (74) [C_5_H_9_^+^]. HRMS (FAB^+^HR,*C*_26_H_28_O_3_) calc. [(*M*+H)^+^]: 399.2899; found: 399.2914.

### Refinement

H atoms were placed in calculated positions, with C—H = 0.95–0.99 Å and were refined as riding, with *U*_iso_= 1.5*U*_eq_ for methyl and 1.2*U*_eq_ for others; the methyl groups were allowed to rotate but not to tip. One methyl group is disordered over two positions in a 0.67:0.33 ratio.

### Crystal data

**Table:**

C_26_H_38_O_3_	*Z* = 2
*M**_r_* = 398.56	*F*(000) = 436
Triclinic, *P*1	*D*_x_ = 1.109 Mg m^−^^3^
Hall symbol: -P 1	Mo *K*α radiation, λ = 0.71073 Å
*a* = 9.534 (2) Å	Cell parameters from 14072 reflections
*b* = 11.753 (3) Å	θ = 3.1–25.0°
*c* = 11.994 (2) Å	µ = 0.07 mm^−^^1^
α = 77.862 (13)°	*T* = 173 K
β = 78.325 (12)°	Block, light yellow
γ = 66.427 (9)°	0.50 × 0.27 × 0.04 mm
*V* = 1193.7 (5) Å^3^	

### Data collection

**Table:**

Nonius KappaCCD diffractometer	1323 reflections with *I* > 2σ(*I*)
Radiation source: fine-focus sealed tube	*R*_int_ = 0.078
graphite	θ_max_ = 25.0°, θ_min_ = 3.1°
Detector resolution: 19 vertical, 18 horizontal pixels mm^-1^	*h* = −11→11
415 frames via ω–rotation (Δω = 2%) and two times 20 s per frame (four sets at different κ–angles) scans	*k* = −12→13
14072 measured reflections	*l* = −13→14
4134 independent reflections	

### Refinement

**Table:**

Refinement on *F*^2^	Primary atom site location: structure-invariant direct methods
Least-squares matrix: full	Secondary atom site location: difference Fourier map
*R*[*F*^2^ > 2σ(*F*^2^)] = 0.036	Hydrogen site location: inferred from neighbouring sites
*wR*(*F*^2^) = 0.112	H-atom parameters constrained
*S* = 1.02	*w* = {exp[6.80(sinθ/λ)^2^]}/[σ^2^(*F*_o_^2^)]
4134 reflections	(Δ/σ)_max_ < 0.001
280 parameters	Δρ_max_ = 0.11 e Å^−^^3^
0 restraints	Δρ_min_ = −0.10 e Å^−^^3^

### Special details

**Table:**

Geometry. All e.s.d.'s (except the e.s.d. in the dihedral angle between two l.s. planes) are estimated using the full covariance matrix. The cell e.s.d.'s are taken into account individually in the estimation of e.s.d.'s in distances, angles and torsion angles; correlations between e.s.d.'s in cell parameters are only used when they are defined by crystal symmetry. An approximate (isotropic) treatment of cell e.s.d.'s is used for estimating e.s.d.'s involving l.s. planes.
Refinement. Refinement of *F*^2^ against ALL reflections. The weighted *R*-factor *wR* and goodness of fit *S* are based on *F*^2^, conventional *R*-factors *R* are based on *F*, with *F* set to zero for negative *F*^2^. The threshold expression of *F*^2^ > σ(*F*^2^) is used only for calculating *R*-factors(gt) *etc*. and is not relevant to the choice of reflections for refinement. *R*-factors based on *F*^2^ are statistically about twice as large as those based on *F*, and *R*- factors based on ALL data will be even larger.

### Fractional atomic coordinates and isotropic or equivalent isotropic displacement parameters (Å^2^)

**Table:**

	*x*	*y*	*z*	*U*_iso_*/*U*_eq_	Occ. (<1)
O1	0.4586 (2)	0.12777 (19)	−0.11632 (17)	0.0438 (6)	
O2	0.2943 (3)	0.5220 (2)	0.00396 (18)	0.0507 (6)	
O3	0.1723 (2)	0.3951 (2)	0.08269 (17)	0.0443 (6)	
C1	0.4204 (4)	0.3126 (3)	−0.0442 (2)	0.0372 (8)	
C2	0.3690 (4)	0.2119 (3)	−0.0635 (2)	0.0386 (8)	
C3	0.2036 (3)	0.2213 (3)	−0.0182 (3)	0.0394 (8)	
C4	0.1504 (4)	0.2817 (3)	0.0889 (3)	0.0392 (8)	
C6	0.2921 (4)	0.4184 (3)	0.0126 (3)	0.0426 (9)	
C7	0.1940 (4)	0.0910 (3)	0.0104 (3)	0.0472 (9)	
H7A	0.2001	0.0604	−0.0620	0.057*	
H7B	0.2831	0.0320	0.0498	0.057*	
C8	0.0452 (4)	0.0929 (3)	0.0869 (3)	0.0530 (10)	
H8A	0.0406	0.0085	0.0988	0.064*	
H8B	−0.0438	0.1524	0.0476	0.064*	
C9	0.0324 (4)	0.1315 (3)	0.2045 (3)	0.0510 (9)	
C10	0.0782 (4)	0.2443 (3)	0.1852 (3)	0.0443 (8)	
H10A	0.0527	0.2909	0.2471	0.053*	
C11	0.4951 (4)	0.3655 (3)	−0.1577 (2)	0.0455 (9)	
H11A	0.5402	0.4215	−0.1405	0.055*	
H11B	0.5809	0.2949	−0.1908	0.055*	
C12	0.3886 (4)	0.4374 (3)	−0.2471 (3)	0.0474 (9)	
H12A	0.3095	0.5145	−0.2298	0.057*	
C13	0.3937 (4)	0.4043 (3)	−0.3474 (3)	0.0541 (10)	
C14	0.5093 (4)	0.2854 (3)	−0.3914 (3)	0.0681 (12)	
H14A	0.5716	0.2319	−0.3308	0.102*	
H14B	0.5769	0.3068	−0.4585	0.102*	
H14C	0.4543	0.2404	−0.4135	0.102*	
C15	0.2786 (4)	0.4871 (3)	−0.4286 (3)	0.0691 (12)	
H15A	0.2106	0.5635	−0.3958	0.104*	
H15B	0.2168	0.4420	−0.4400	0.104*	
H15C	0.3338	0.5097	−0.5027	0.104*	
C21	0.5472 (3)	0.2483 (3)	0.0391 (2)	0.0411 (8)	
H21A	0.6433	0.1969	−0.0051	0.049*	
H21B	0.5685	0.3152	0.0624	0.049*	
C22	0.5124 (3)	0.1669 (3)	0.1455 (3)	0.0399 (8)	
H22A	0.5189	0.0868	0.1354	0.048*	
C23	0.4738 (4)	0.1926 (3)	0.2516 (3)	0.0417 (8)	
C24	0.4434 (4)	0.3177 (3)	0.2852 (3)	0.0564 (10)	
H24A	0.4566	0.3760	0.2161	0.085*	
H24B	0.5163	0.3071	0.3372	0.085*	
H24C	0.3376	0.3513	0.3242	0.085*	
C25	0.4543 (4)	0.0965 (3)	0.3513 (3)	0.0584 (10)	
H25A	0.4790	0.0171	0.3236	0.088*	
H25B	0.3473	0.1258	0.3888	0.088*	
H25C	0.5239	0.0839	0.4066	0.088*	
C31	0.1015 (4)	0.3067 (3)	−0.1133 (2)	0.0476 (9)	
H31A	−0.0082	0.3225	−0.0828	0.057*	
H31B	0.1141	0.3885	−0.1306	0.057*	
C32	0.1389 (4)	0.2524 (3)	−0.2233 (3)	0.0529 (10)	
H32A	0.2407	0.1933	−0.2390	0.064*	
C33	0.0465 (4)	0.2776 (3)	−0.2995 (3)	0.0473 (9)	
C34	0.0995 (4)	0.2205 (3)	−0.4097 (3)	0.0587 (10)	
H34A	0.2051	0.1584	−0.4084	0.088*	
H34B	0.0970	0.2866	−0.4754	0.088*	
H34C	0.0307	0.1795	−0.4166	0.088*	
C35	−0.1086 (10)	0.3876 (7)	−0.2954 (8)	0.060 (2)	0.67
H35A	−0.1338	0.4175	−0.2207	0.091*	0.67
H35B	−0.1890	0.3602	−0.3059	0.091*	0.67
H35C	−0.1025	0.4557	−0.3569	0.091*	0.67
C35'	−0.124 (2)	0.3261 (15)	−0.273 (2)	0.074 (6)	0.33
H35D	−0.1603	0.2575	−0.2658	0.111*	0.33
H35E	−0.1692	0.3918	−0.3352	0.111*	0.33
H35F	−0.1550	0.3610	−0.2005	0.111*	0.33
C41	−0.1364 (4)	0.1687 (4)	0.2621 (3)	0.0694 (11)	
H41A	−0.1462	0.1968	0.3357	0.104*	
H41B	−0.1669	0.0961	0.2756	0.104*	
H41C	−0.2035	0.2369	0.2117	0.104*	
C42	0.1373 (4)	0.0236 (3)	0.2825 (3)	0.0659 (11)	
H42A	0.2441	−0.0021	0.2444	0.099*	
H42B	0.1039	−0.0475	0.2974	0.099*	
H42C	0.1311	0.0515	0.3554	0.099*	

### Atomic displacement parameters (Å^2^)

**Table:**

	*U*^11^	*U*^22^	*U*^33^	*U*^12^	*U*^13^	*U*^23^
O1	0.0398 (14)	0.0366 (14)	0.0521 (14)	−0.0077 (11)	−0.0051 (11)	−0.0148 (11)
O2	0.0497 (15)	0.0371 (15)	0.0653 (16)	−0.0125 (12)	−0.0087 (12)	−0.0139 (12)
O3	0.0433 (15)	0.0355 (14)	0.0523 (14)	−0.0116 (12)	−0.0008 (12)	−0.0143 (11)
C1	0.038 (2)	0.036 (2)	0.0391 (18)	−0.0108 (17)	−0.0070 (16)	−0.0122 (16)
C2	0.044 (2)	0.034 (2)	0.0362 (19)	−0.0085 (18)	−0.0121 (16)	−0.0057 (16)
C3	0.035 (2)	0.036 (2)	0.0446 (19)	−0.0058 (17)	−0.0098 (16)	−0.0094 (16)
C4	0.037 (2)	0.033 (2)	0.048 (2)	−0.0101 (17)	−0.0093 (17)	−0.0093 (17)
C6	0.044 (2)	0.037 (2)	0.048 (2)	−0.0129 (18)	−0.0111 (18)	−0.0096 (17)
C7	0.040 (2)	0.040 (2)	0.064 (2)	−0.0118 (17)	−0.0118 (18)	−0.0128 (18)
C8	0.040 (2)	0.047 (2)	0.077 (3)	−0.0190 (18)	−0.0073 (19)	−0.015 (2)
C9	0.039 (2)	0.053 (2)	0.066 (2)	−0.0235 (19)	−0.0032 (19)	−0.010 (2)
C10	0.036 (2)	0.046 (2)	0.046 (2)	−0.0101 (17)	−0.0059 (17)	−0.0082 (17)
C11	0.049 (2)	0.040 (2)	0.047 (2)	−0.0147 (18)	−0.0057 (17)	−0.0087 (17)
C12	0.058 (2)	0.036 (2)	0.046 (2)	−0.0153 (18)	−0.0088 (18)	−0.0029 (17)
C13	0.073 (3)	0.048 (2)	0.046 (2)	−0.028 (2)	−0.0129 (19)	−0.0014 (18)
C14	0.092 (3)	0.059 (3)	0.053 (2)	−0.025 (2)	−0.008 (2)	−0.017 (2)
C15	0.093 (3)	0.062 (3)	0.054 (2)	−0.026 (2)	−0.031 (2)	0.004 (2)
C21	0.036 (2)	0.042 (2)	0.046 (2)	−0.0108 (17)	−0.0068 (16)	−0.0115 (17)
C22	0.037 (2)	0.035 (2)	0.045 (2)	−0.0099 (16)	−0.0079 (16)	−0.0056 (17)
C23	0.039 (2)	0.041 (2)	0.043 (2)	−0.0098 (17)	−0.0073 (16)	−0.0086 (17)
C24	0.065 (3)	0.054 (2)	0.051 (2)	−0.019 (2)	−0.0064 (19)	−0.0158 (18)
C25	0.063 (3)	0.058 (3)	0.051 (2)	−0.019 (2)	−0.0108 (19)	−0.0045 (19)
C31	0.040 (2)	0.048 (2)	0.051 (2)	−0.0079 (17)	−0.0101 (17)	−0.0118 (18)
C32	0.036 (2)	0.064 (3)	0.053 (2)	−0.0066 (18)	−0.0073 (18)	−0.0204 (19)
C33	0.040 (2)	0.054 (2)	0.049 (2)	−0.0168 (18)	−0.0026 (17)	−0.0143 (18)
C34	0.058 (3)	0.066 (3)	0.052 (2)	−0.021 (2)	−0.0042 (19)	−0.016 (2)
C35	0.051 (5)	0.072 (7)	0.054 (4)	−0.009 (5)	−0.016 (3)	−0.018 (5)
C35'	0.055 (10)	0.060 (13)	0.120 (17)	−0.015 (10)	−0.024 (9)	−0.043 (12)
C41	0.053 (3)	0.076 (3)	0.089 (3)	−0.034 (2)	0.005 (2)	−0.025 (2)
C42	0.061 (3)	0.060 (3)	0.072 (3)	−0.024 (2)	−0.010 (2)	0.006 (2)

### Geometric parameters (Å, °)

**Table:**

O1—C2	1.216 (3)	C21—C22	1.491 (4)
O2—C6	1.207 (3)	C21—H21A	0.9900
O3—C6	1.361 (4)	C21—H21B	0.9900
O3—C4	1.413 (3)	C22—C23	1.316 (4)
C1—C2	1.523 (4)	C22—H22A	0.9500
C1—C6	1.519 (4)	C23—C25	1.499 (4)
C1—C11	1.534 (4)	C23—C24	1.506 (4)
C1—C21	1.572 (4)	C24—H24A	0.9800
C2—C3	1.527 (4)	C24—H24B	0.9800
C3—C4	1.498 (4)	C24—H24C	0.9800
C3—C7	1.533 (4)	C25—H25A	0.9800
C3—C31	1.563 (4)	C25—H25B	0.9800
C4—C10	1.307 (4)	C25—H25C	0.9800
C7—C8	1.520 (4)	C31—C32	1.502 (4)
C7—H7A	0.9900	C31—H31A	0.9900
C7—H7B	0.9900	C31—H31B	0.9900
C8—C9	1.539 (4)	C32—C33	1.305 (4)
C8—H8A	0.9900	C32—H32A	0.9500
C8—H8B	0.9900	C33—C35	1.526 (9)
C9—C10	1.514 (4)	C33—C34	1.511 (4)
C9—C42	1.532 (4)	C33—C35'	1.48 (2)
C9—C41	1.541 (4)	C34—H34A	0.9800
C10—H10A	0.9500	C34—H34B	0.9800
C11—C12	1.506 (4)	C34—H34C	0.9800
C11—H11A	0.9900	C35—H35A	0.9800
C11—H11B	0.9900	C35—H35B	0.9800
C12—C13	1.327 (4)	C35—H35C	0.9800
C12—H12A	0.9500	C35'—H35D	0.9800
C13—C15	1.510 (4)	C35'—H35E	0.9800
C13—C14	1.513 (5)	C35'—H35F	0.9800
C14—H14A	0.9800	C41—H41A	0.9800
C14—H14B	0.9800	C41—H41B	0.9800
C14—H14C	0.9800	C41—H41C	0.9800
C15—H15A	0.9800	C42—H42A	0.9800
C15—H15B	0.9800	C42—H42B	0.9800
C15—H15C	0.9800	C42—H42C	0.9800
			
C6—O3—C4	121.5 (3)	H15B—C15—H15C	109.5
C2—C1—C6	113.8 (3)	C22—C21—C1	117.3 (3)
C2—C1—C11	110.5 (2)	C22—C21—H21A	108.0
C6—C1—C11	110.3 (3)	C1—C21—H21A	108.0
C2—C1—C21	107.5 (2)	C22—C21—H21B	108.0
C6—C1—C21	106.9 (2)	C1—C21—H21B	108.0
C11—C1—C21	107.5 (2)	H21A—C21—H21B	107.2
O1—C2—C1	119.7 (3)	C23—C22—C21	127.8 (3)
O1—C2—C3	121.5 (3)	C23—C22—H22A	116.1
C1—C2—C3	118.8 (3)	C21—C22—H22A	116.1
C4—C3—C2	109.1 (2)	C22—C23—C25	121.5 (3)
C4—C3—C7	108.7 (3)	C22—C23—C24	124.6 (3)
C2—C3—C7	110.9 (2)	C25—C23—C24	113.9 (3)
C4—C3—C31	109.3 (2)	C23—C24—H24A	109.5
C2—C3—C31	106.9 (3)	C23—C24—H24B	109.5
C7—C3—C31	111.7 (2)	H24A—C24—H24B	109.5
C10—C4—O3	116.7 (3)	C23—C24—H24C	109.5
C10—C4—C3	126.7 (3)	H24A—C24—H24C	109.5
O3—C4—C3	116.5 (3)	H24B—C24—H24C	109.5
O2—C6—O3	117.3 (3)	C23—C25—H25A	109.5
O2—C6—C1	123.3 (3)	C23—C25—H25B	109.5
O3—C6—C1	119.2 (3)	H25A—C25—H25B	109.5
C8—C7—C3	111.9 (3)	C23—C25—H25C	109.5
C8—C7—H7A	109.2	H25A—C25—H25C	109.5
C3—C7—H7A	109.2	H25B—C25—H25C	109.5
C8—C7—H7B	109.2	C32—C31—C3	114.1 (3)
C3—C7—H7B	109.2	C32—C31—H31A	108.7
H7A—C7—H7B	107.9	C3—C31—H31A	108.7
C7—C8—C9	112.3 (2)	C32—C31—H31B	108.7
C7—C8—H8A	109.2	C3—C31—H31B	108.7
C9—C8—H8A	109.2	H31A—C31—H31B	107.6
C7—C8—H8B	109.2	C33—C32—C31	127.0 (3)
C9—C8—H8B	109.2	C33—C32—H32A	116.5
H8A—C8—H8B	107.9	C31—C32—H32A	116.5
C10—C9—C42	110.0 (3)	C32—C33—C35	121.1 (4)
C10—C9—C41	108.8 (3)	C32—C33—C34	122.2 (3)
C42—C9—C41	109.6 (3)	C35—C33—C34	115.4 (4)
C10—C9—C8	108.4 (3)	C32—C33—C35'	123.6 (9)
C42—C9—C8	110.9 (3)	C34—C33—C35'	111.2 (8)
C41—C9—C8	109.1 (3)	C33—C34—H34A	109.5
C4—C10—C9	124.3 (3)	C33—C34—H34B	109.5
C4—C10—H10A	117.8	H34A—C34—H34B	109.5
C9—C10—H10A	117.8	C33—C34—H34C	109.5
C12—C11—C1	115.5 (3)	H34A—C34—H34C	109.5
C12—C11—H11A	108.4	H34B—C34—H34C	109.5
C1—C11—H11A	108.4	C33—C35—H35A	109.5
C12—C11—H11B	108.4	C33—C35—H35B	109.5
C1—C11—H11B	108.4	C33—C35—H35C	109.5
H11A—C11—H11B	107.5	C33—C35'—H35D	109.5
C13—C12—C11	126.8 (3)	C33—C35'—H35E	109.5
C13—C12—H12A	116.6	H35D—C35'—H35E	109.5
C11—C12—H12A	116.6	C33—C35'—H35F	109.5
C12—C13—C15	120.6 (3)	H35D—C35'—H35F	109.5
C12—C13—C14	125.2 (3)	H35E—C35'—H35F	109.5
C15—C13—C14	114.2 (3)	C9—C41—H41A	109.5
C13—C14—H14A	109.5	C9—C41—H41B	109.5
C13—C14—H14B	109.5	H41A—C41—H41B	109.5
H14A—C14—H14B	109.5	C9—C41—H41C	109.5
C13—C14—H14C	109.5	H41A—C41—H41C	109.5
H14A—C14—H14C	109.5	H41B—C41—H41C	109.5
H14B—C14—H14C	109.5	C9—C42—H42A	109.5
C13—C15—H15A	109.5	C9—C42—H42B	109.5
C13—C15—H15B	109.5	H42A—C42—H42B	109.5
H15A—C15—H15B	109.5	C9—C42—H42C	109.5
C13—C15—H15C	109.5	H42A—C42—H42C	109.5
H15A—C15—H15C	109.5	H42B—C42—H42C	109.5
			
C6—C1—C2—O1	174.3 (3)	C31—C3—C7—C8	−77.5 (3)
C11—C1—C2—O1	49.6 (4)	C3—C7—C8—C9	−62.6 (4)
C21—C1—C2—O1	−67.4 (3)	C7—C8—C9—C10	45.2 (4)
C6—C1—C2—C3	−4.2 (4)	C7—C8—C9—C42	−75.7 (4)
C11—C1—C2—C3	−128.9 (3)	C7—C8—C9—C41	163.6 (3)
C21—C1—C2—C3	114.1 (3)	O3—C4—C10—C9	174.7 (3)
O1—C2—C3—C4	149.8 (3)	C3—C4—C10—C9	−1.0 (5)
C1—C2—C3—C4	−31.7 (4)	C42—C9—C10—C4	106.6 (4)
O1—C2—C3—C7	30.1 (4)	C41—C9—C10—C4	−133.3 (3)
C1—C2—C3—C7	−151.5 (3)	C8—C9—C10—C4	−14.8 (4)
O1—C2—C3—C31	−92.0 (3)	C2—C1—C11—C12	66.1 (3)
C1—C2—C3—C31	86.5 (3)	C6—C1—C11—C12	−60.6 (3)
C6—O3—C4—C10	153.2 (3)	C21—C1—C11—C12	−176.8 (3)
C6—O3—C4—C3	−30.6 (4)	C1—C11—C12—C13	−113.0 (4)
C2—C3—C4—C10	−134.2 (3)	C11—C12—C13—C15	−179.7 (3)
C7—C3—C4—C10	−13.0 (4)	C11—C12—C13—C14	0.3 (6)
C31—C3—C4—C10	109.2 (4)	C2—C1—C21—C22	−48.5 (4)
C2—C3—C4—O3	50.1 (3)	C6—C1—C21—C22	74.2 (3)
C7—C3—C4—O3	171.2 (2)	C11—C1—C21—C22	−167.4 (3)
C31—C3—C4—O3	−66.6 (3)	C1—C21—C22—C23	−104.8 (4)
C4—O3—C6—O2	173.1 (3)	C21—C22—C23—C25	−175.1 (3)
C4—O3—C6—C1	−10.8 (4)	C21—C22—C23—C24	5.7 (5)
C2—C1—C6—O2	−156.6 (3)	C4—C3—C31—C32	−177.0 (3)
C11—C1—C6—O2	−31.8 (4)	C2—C3—C31—C32	64.9 (3)
C21—C1—C6—O2	84.8 (4)	C7—C3—C31—C32	−56.6 (4)
C2—C1—C6—O3	27.5 (4)	C3—C31—C32—C33	155.0 (3)
C11—C1—C6—O3	152.3 (2)	C31—C32—C33—C35	11.5 (7)
C21—C1—C6—O3	−91.1 (3)	C31—C32—C33—C34	177.5 (3)
C4—C3—C7—C8	43.2 (3)	C31—C32—C33—C35'	−24.0 (9)
C2—C3—C7—C8	163.2 (2)		
